# Alterations of mitochondrial dynamics allow retrograde propagation of locally initiated axonal insults

**DOI:** 10.1038/srep32777

**Published:** 2016-09-08

**Authors:** Benjamin Lassus, Sebastien Magifico, Sandra Pignon, Pascale Belenguer, Marie-Christine Miquel, Jean-Michel Peyrin

**Affiliations:** 1 CNRS UMR 8256, Biological Adaptation and Ageing, Paris, 75005, France; 2 Sorbonne Universités, UPMC, Institut de Biologie Paris-Seine, Paris, 75005, France; 3 CNRS UMR 5169 Research Center on Animal Cognition, Center for Integrative Biology, Toulouse University, Université Toulouse 3 Paul Sabatier, 31400, France

## Abstract

In chronic neurodegenerative syndromes, neurons progressively die through a generalized retraction pattern triggering retrograde axonal degeneration toward the cell bodies, which molecular mechanisms remain elusive. Recent observations suggest that direct activation of pro-apoptotic signaling in axons triggers local degenerative events associated with early alteration of axonal mitochondrial dynamics. This raises the question of the role of mitochondrial dynamics on both axonal vulnerability stress and their implication in the spreading of damages toward unchallenged parts of the neuron. Here, using microfluidic chambers, we assessed the consequences of interfering with OPA1 and DRP1 proteins on axonal degeneration induced by local application of rotenone. We found that pharmacological inhibition of mitochondrial fission prevented axonal damage induced by rotenone, in low glucose conditions. While alteration of mitochondrial dynamics *per se* did not lead to spontaneous axonal degeneration, it dramatically enhanced axonal vulnerability to rotenone, which had no effect in normal glucose conditions, and promoted retrograde spreading of axonal degeneration toward the cell body. Altogether, our results suggest a mitochondrial priming effect in axons as a key process of axonal degeneration. In the context of neurodegenerative diseases, like Parkinson’s and Alzheimer’s, mitochondria fragmentation could hasten neuronal death and initiate spatial dispersion of locally induced degenerative events.

Axonal and synaptic degeneration are key processes in neurodegenerative diseases. Neurons degenerate through a protracted Dying-Back pattern, sequentially it involves collapse of synaptic ends, dismantling of axonal tracts and, ultimately, degeneration of the cell body^1^. While the mechanisms involved in neuronal soma destruction have been extensively studied, the molecular cues leading to axonal degeneration remain elusive. Seminal studies on Wallerian Degeneration and Wallerian Degeneration Slow (WLD(s)) spontaneous mutant mice, have suggested that axons and somas degenerate through distinct mechanisms^2^. Indeed, upon axotomy, while retrograde degeneration of the axons towards the cell body entails apoptotic signaling, the destruction of the distal part of axons implicates an orchestrated process involving important modifications of NAD^+^-associated signaling pathways^3^. Once proposed to be mediated through nuclear production of NAD^+ ^4, increasing evidence indicates that cytoplasmic or even mitochondrial production of NAD^+^ mediates a strong axo-protective effect^5^,^6^. Consistent with these notions, axonal transport conveys NAD+ producing enzymes to the distal part of the axons^7^,^8^. Moreover, axotomy, peripheral microtubule destabilization or apoptosis signaling have all been shown to trigger axonal NAD^+^ depletion associated with mitochondrial transport impairment and mitochondrial dysfunctions such as mitochondrial transition Pore (mPTP) opening in axonal endings^9^. We and others have shown that NAD^+^ cross talks with local apoptotic pathways and apoptosome in axons^10^,^11^, partially through mitochondrial SirT3 activation^12^,^13^. This is in line with evidence showing that effectors of the pro-apoptotic modules control degenerative processes in axons triggered during the neurodevelopmental phase^14^,^15^,^16^ a period associated with extreme vulnerability of neurons toward apoptosis^17^,^18^ and that the modality of apoptotic modules activation is itself compartmentalized^19^. Therefore, several lines of evidence point toward axonal mitochondria playing a crucial role in gate-keeping axonal vulnerability toward stressors which involve subtle adjustments of mitochondrial functions. Mitochondrial dynamics and quality control regulate these functions, including local energy supply and sequestration of pro-apoptotic factors.

Mitochondria are complex versatile organelles, forming networks that are constantly remodeled through significant fusion and fission events, which are tightly controlled by several key proteins with GTPase activity. Dynamin-related protein 1 (DRP1) manages fission while Mitofusins (MFN1, MFN2) and OPA1 handle fusion of mitochondria^20^,^21^,^22^,^23^. This dynamics is greatly modified during apoptosis, and fission of the mitochondrial network is considered an early event of apoptosis^24^,^25^,^26^. Consequently, several studies have shown that an increase of mitochondrial fission, through DRP1 overexpression or by Mitofusins and/or OPA1 inhibition, enhances cell vulnerability toward apoptosis, or sensitizes cells to stress^24^,^27^,^28^,^29^, particularly neurons^30^,^31^. Moreover, MFN2 is necessary for axonal mitochondrial transport and positioning^32^,^33^, and alteration of mitochondrial fission through DRP1 inactivation or OPA1 inactivation leads to abnormal mitochondria positioning in neurites^34^,^35^. Alteration of mitochondrial dynamics has been associated to the early stage of neurodegenerative conditions^20^,^36^,^37^; and inhibiting DRP1 proved to be a promising strategy in order to delay neuronal demise^31^,^38^. However, the exact role of mitochondrial dynamics in axonal susceptibility and/or resistance to direct focalized insults remains a poorly documented question. While inhibition of MFN2 was reported to lead to spontaneous axonal degeneration in peripheral neurons^33^, direct axonal insults such as abnormal calcium influx, or pro inflammatory conditions were shown to trigger local mitochondrial and axonal alteration *in vitro*^30^. Importantly, these alterations were demonstrated to be reversible^39^. While this suggests that the spread of mitochondrial alteration may be buffered by unchallenged parts of the axons, the question remains whether damaged fragmented mitochondria can convey dysfunction/pro-apoptotic signals to distant, unchallenged areas of the neuron.

Here, we aim to study the consequences of mitochondrial dynamics alteration on axonal vulnerability to axonal pro-apoptotic stress. Using primary neurons in compartmented culture devices, we applied rotenone, a mitochondrial stressor, on the distal parts of axons. We evaluated the influence of mitochondrial dynamics alterations on axonal vulnerability through pharmacological inhibition of mitochondrial fission with Mdivi-1, as well as overexpression of wild type or mutant DRP1 or OPA1 proteins. Finally, this paradigm allowed us to analyze the impact of mitochondrial fission on the retrograde spreading of apoptotic signals along axons.

## Results

### Genetic manipulation of mitochondrial dynamics does not trigger spontaneous axonal degeneration

**Figure 1:**
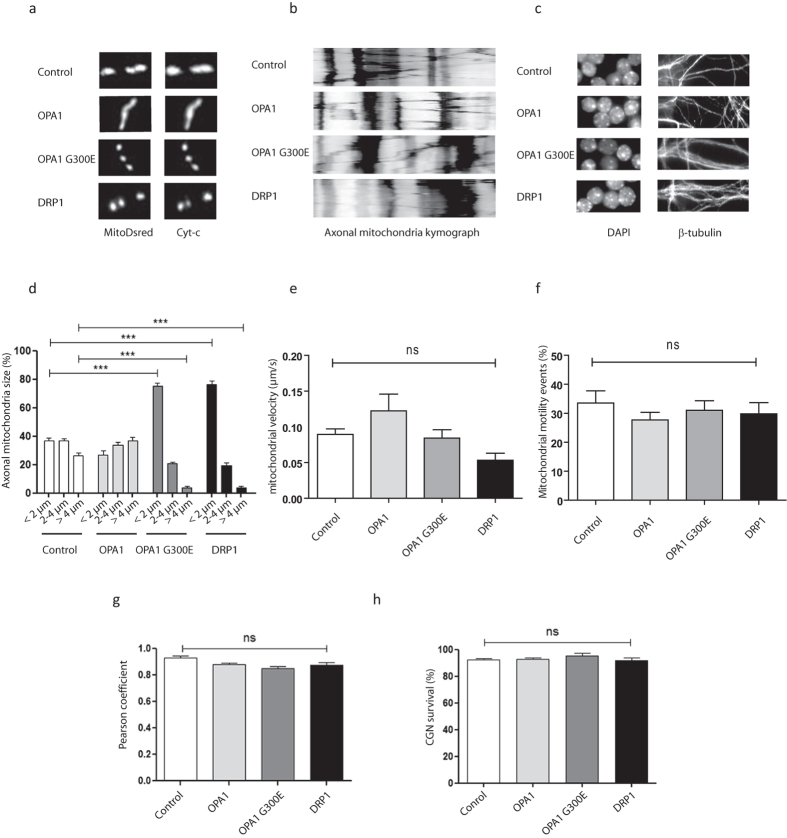
Genetic manipulation of mitochondrial dynamics does not trigger spontaneous axonal degeneration and cell death. (**a**) Morphology of mitochondria in distal CGN axons 10 days after co-transfection with control, OPA1, OPA1^G300E^ or DRP1 and MitoDsRed vectors, illustrated by representative images of the most abundant forms for each condition. Mitochondria were visualized with Mito-DsRed fluorescence and immunostaining of cytochrome *c* (Cyt-c). (**b**) Kymograph analysis of mitochondrial mobility in the axonal, chamber in control condition and after overexpression of OPA1, OPA1^G300E^ or DRP1. (**c**) Illustration of DAPI staining (left) and beta3-tubulin immunostaining (right) of GCN 10 DIV after transfection of control, OPA1, OPA1^G300E^ or DRP1 vectors. (**d**) Repartition of mitochondrial morphology observed upon overexpression of OPA1, OPA1^G300E^ or DRP1, into three main classes: i) fragmented, under or equal at 2 μm long, ii) filamentous, between 2 and 4 μm, and iii) hyper-filamentous, above 4 μm. (**e**) Quantification of mitochondrial velocity upon OPA1, OPA1G300E and DRP1 overexpression, in distal axonal segments. (**f**) Quantification of mitochondrial motility upon OPA1, OPA1G300E and DRP1 overexpression, in distal axonal segments. (**g**) Co-localization of Mito-DsRed and cytochrome *c* signals assessed by a Pearson coefficient analysis. (**h**) Quantification of CGN survival assessed by nuclear (DAPI) staining. Each experiment was conducted 3 times independently in triplicates and data were analyzed using ANOVA statistical method.

Mitochondrial fission is an early event preceding axonal tubulin dismantlement after either somatic or axonal insults. In previous studies we showed that axo-protective molecules impaired both mitochondrial fission and axonal degeneration processes^13^,^40^. Here, in order to assess whether fission could be a cause or a consequence of ongoing axonal degeneration, we investigated the direct role of mitochondrial dynamics on spontaneous axonal degeneration. We used a previously developed model of Cerebellar Granule Neurons (CGN) grown in microfluidic chambers which allowed the compartmentalization of axons and somas in two separate chambers. Because mitochondrial fragmentation can be a result an impaired fusion and/or an increase in fission, we used genetic manipulation of two key proteins that control mitochondrial dynamics: (1) the profusion protein OPA1 and (2) the pro-fission protein DRP1. Prior to seeding in the microfluidic devices, CGN neurons were electroporated with constructs encoding either wild type OPA1, or OPA1^G330E^, a dominant negative mutant of OPA1^41^, or wild type DRP1. Co-transfection with the MitoDsRed expression vector allowed for direct visualization of mitochondrial morphology^35^. Quantitative analysis of axonal mitochondrial morphology 10 days after transfection showed that in control condition, small fissionned mitochondria (≤2 μm long), represented approximately 40% of the mitochondrial population, while the remaining ≈60% consisted of filamentous (2–4 μm) and hyper-filamentous (≥4 μm) mitochondria (Fig. 1a,d). Overexpression of OPA1 did not modify these percentages to statistical significance, although slightly displacing the repartition toward longer forms. As expected, forcing fission by inactivation of OPA1 through overexpression of OPA1^G300E^ or by overexpression of the pro-fission protein DRP1 led to a dramatic increase in fragmented mitochondria that accounted for almost 70% of the total mitochondria (Fig. 1a,d). The impact of these mitochondrial dynamics modulations on mitochondrial mobility was then studied through kymograph analysis (Fig. 1b). In the control condition, approximately 80% of mitochondria are immobile and approximately 30% have anterograde or retrograde mobility with an average speed of 0.1 μm.s^−1^ (Fig. 1e,f). Neither OPA1^G300E^ nor DRP1 overexpression did modify the percentage of motile mitochondria or the average speed of mitochondria (Fig. 1e,f). Accordingly, the repartition of fragmented mitochondria in OPA1^G300E^ or DRP1 conditions was homogeneous in both the somato-dendritic compartment and axonal endings of CGN, and no major change was observed as compared to the control neurons (data not shown). To test whether altered fragmented mitochondria could be engaged in the apoptotic pathway, the distribution of cytochrome *c* was compared to MitoDsRed, allowing detection of potentially deleterious cytochrome *c* leaks from mitochondria. As shown in Fig. 1a,g, the MitoDsRed and cytochrome *c* signals were co-localized, even in fragmented mitochondria following OPA1^G300E^ or DRP1 overexpression, thus indicating that mitochondria were not engaged in a pro-apoptotic process. Furthermore, analysis of CGN morphology showed that none of the conditions impacted neuronal survival as assessed by nuclear condensation in the somatic chamber and axonal beta3-tubulin staining (Fig. 1c,h). In conclusion, our data show that increasing mitochondrial fission through overexpression of OPA1^G300E^ or DRP1, results in an increase to 80% of fragmented mitochondria in both somato-dendritic and axonal compartments, as compared to 40% in control conditions. However this did not significantly impact axonal fate nor lead to spontaneous activation of apoptotic pathways in CGN.

### Local pharmacological inhibition of mitochondrial fission prevented axonal damage

**Figure 2:**
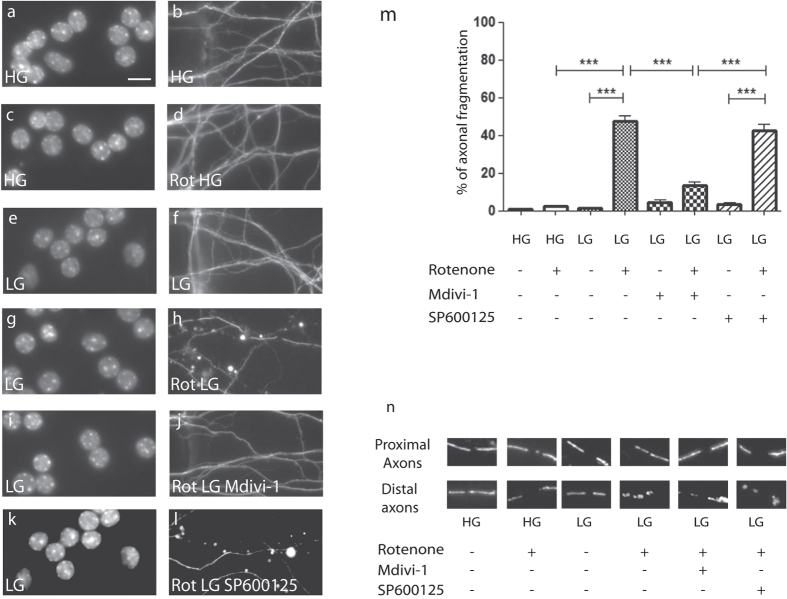
Local pharmacological inhibition of mitochondrial fission prevented axonal damage. (**a–l**) Paired morphology panel of DIV10 CGN in microfluidic chambers. The somatic chamber was probed with Hoechst nuclear staining (**a,c,e,g,i,k**) and axonal endings in the axonal chamber immuno-stained with beta3-tubulin (**b,d,f,h,j,l**). (**a,b**), 10 DIV CGN grown in high glucose (HG) conditions. (**c,d**) Application of axonal rotenone (Rot HG) in the axonal chamber did not trigger axonal degeneration in HG condition after 24 hours of treatment. (**e,f**) Low glucose (LG) condition was innocuous. (**g,h**) Application of axonal rotenone in LG triggered axonal degeneration. (**i,j**) Application of 10 μM Mdivi-1 on axons inhibited the action of rotenone, but 10 μM SP600125 did not (**k,l**). (Scale bar: 10 μm). (**m**) Quantification of axonal degeneration. Each experiment was conducted at 3 times independently in triplicates and data were analyzed using ANOVA statistical method. (**n**) Representative images of both proximal (somatic chambers) and distal (axonal chamber) axonal mitochondrial morphology upon TOM20 immunostaining after rotenone insult and pharmacological blockade.

While increasing mitochondrial fission *per se* did not lead to spontaneous axonal degeneration, mitochondrial fission could sensitize axons toward stressors such as rotenone. This mitochondrial poison promotes neuronal apoptosis and primarily targets the Complex I of the mitochondrial respiratory chain, triggers mitochondrial ROS production. Due to microfluidic barriers and axons occluding the micro channels, molecular diffusion between axonal and somatic chambers is highly limited^42^,^43^. Using this to our advantage, we tested whether mitochondrial fission modifies the vulnerability of axons toward susceptibility to pro apoptotic stressor. Rotenone was selectively applied to CGN axonal compartments in order to initiate local degenerative events. Axonal application of up to 5 μM of rotenone in High Glucose (HG) condition had no visible effect on both axonal mitochondrial morphology and axonal degeneration (Fig. 2a–d,m,n). However, as previously observed^13^, in glycolytically impaired conditions (i.e. in Low Glucose (LG) conditions), rotenone induced extensive axonal mitochondrial fission and axonal degeneration (Fig. 2e–h,m,n). A striking observation was that while rotenone treatment of axonal endings in glycolytically impaired conditions triggers complete axonal degeneration in the distal (treated) chambers, there is almost no observable retrograde spreading of the axonal damage toward the cell body, as assessed by nuclear integrity staining after 24 hours (Fig. 2g). Yet, careful examination and morphological analysis of GFP-transfected at a later time point (axons treated for 48 hours) showed evidence of a slow and partial retrograde degeneration (Sup Fig. 1).

We then evaluated the efficiency of pharmacological inhibition of mitochondrial fission in impeding rotenone-induced axonal degeneration. While application of SP600125, a JNK inhibitor showed no protective effect (Fig. 2n,k–l,m), application of Mdivi-1, an inhibitor of DRP1^44^,^45^, in the axonal chamber prevented both mitochondrial fragmentation and axonal degeneration induced by rotenone in LG medium (Fig. 2n,i,j,m). Cumulatively, these data demonstrate that preventing mitochondrial alteration at the DRP1 level is sufficient to prevent rotenone-induced axonal degeneration.

### Increasing fission enhanced axonal vulnerability to rotenone and restricts retrograde spreading toward the cell body

**Figure 3:**
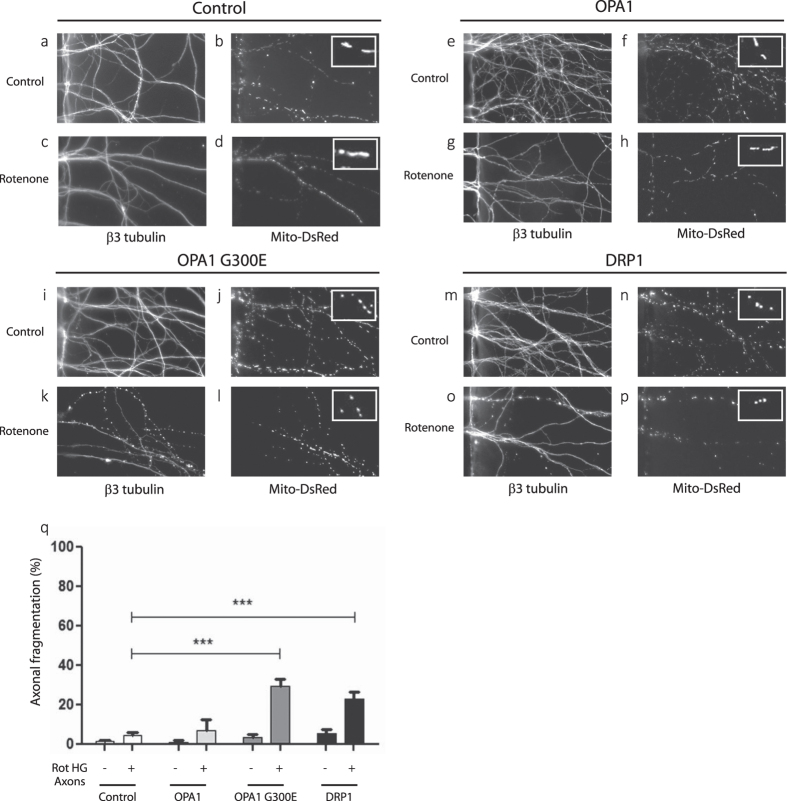
Genetic manipulation of mitochondrial dynamics affects axonal vulnerability to rotenone. (**a–p**) Representative fluorescence images of axons from CGN, 10 days after co-transfection with 1) MitoDsRed vectors and 2) control, OPA1, OPA1^G300E^ or DRP1 vectors. Axonal endings were visualized after β3-tubulin immunostaining (left panels) and mitochondria by Mito-DsRed fluorescence (right panels). CGN were grown in HG condition and axons were treated with vehicle (Control) (**a,b,e,f,i,j,m,n**) or 5 μM rotenone (**c,d,g,h,k,l,o,p**) for 24 hours. (**q**) Bar graph of the quantification of axonal degeneration in all conditions. Each experiment was conducted 3 times independently in triplicates and data were analyzed using ANOVA statistical method. Insert: Representative images of axonal mitochondria upon the various conditions studied.

Alteration of mitochondrial dynamics by genetic means may lead to subtle axonal alterations in mitochondrial physiology, which although non-lethal *per se*, have been proposed to sensitize cells to further stress^28^,^41^. In order to assess whether fragmented axonal mitochondria are associated with increased vulnerability to axonal rotenone, we exposed the axons of OPA1-, OPA1^G300E^- and DRP1-transfected neurons to 5 μM rotenone in HG cell culture medium, a sub-threshold condition in which rotenone has no deleterious effect. As shown in Fig. 3a–d,q, in HG medium, the expression of control and Mito-DsRed vectors, alone or in combination with rotenone (5 μM) axonal application, had no effect on mitochondrial morphology or axonal integrity. Overexpression of OPA1 did not modify axonal integrity or the overall mitochondrial morphology with or without rotenone (Fig. 3e–h). However, application of rotenone in HG medium of both OPA1^G300E^ (Fig. 3i–l) and DRP1 (Fig. 3m–p) treated axons resulted in 30% axonal fragmentation after a 24 h treatment (Fig. 3q). It can be concluded that mitochondrial fission significantly enhances axonal vulnerability to rotenone and allows to overcome the inhibitory effect of high glycolytic environment. Interestingly, at time points where axons show no sign of degeneration (6h), rotenone treatment of axonal segment triggered a decrease in both mitochondria movement (from 33% to 12% ) and speed (0.95 to 0.04 μm/s^−1^). This effect was even more pronounced under DRP1 overexpression condition where mitochondria virtually halted in the treated segments (motility drops from 30% to 1.8% and speed from 0.05 to 0.015 μm/s^−1^).

**Figure 4:**
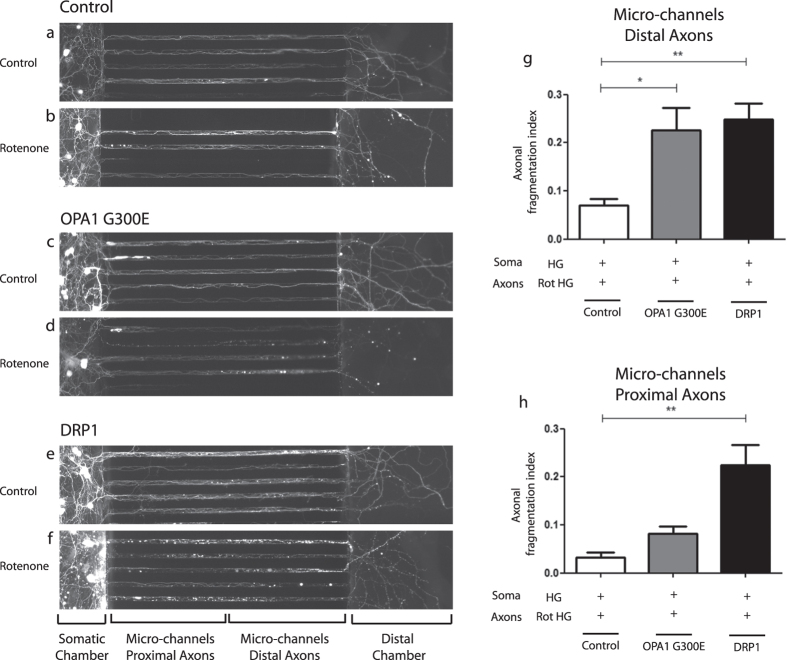
Genetic manipulation of mitochondrial dynamics promotes retrograde spreading of axonal degeneration toward the cell body after local rotenone insult. (**a–f**) Representative fluorescence images (mosaic tiled reconstruction into one image) of neurons from CGN, 10 days after co-transfection with control, OPA1, OPA1^G300E^ or DRP1 and GFP vectors seeded in microfluidic chambers. CGN were grown in high glucose (HG) condition and axons were treated with vehicle (Control) (**a,c,e**) or 5 μM rotenone (**b,d,f**) for 24 hours. Note the formation of axonal blebbing reminiscent of axonal degeneration inside the micro-channel area. (**g,h**) quantification of the axonal degeneration index in the distal (**g**) and proximal parts (**h**) of the micro-channel areas. Each experiment was conducted at least 3 times independently in triplicates and data were analyzed using ANOVA statistical method.

As described in Fig. 2, axonal application of rotenone triggers destruction of the treated axonal endings associated with a minor and very slow retrograde degeneration profile inside the micro-channel area. A plausible scenario would be that mitochondria lying in the unchallenged parts of the axons may act as buffer, limiting the retrograde spread of locally imitated insults. In order to assess whether mitochondrial fission may modify retrograde degeneration, together with OPA1^G300E^ or DRP1 plasmids CGN neurons were co-transfected with a GFP encoding vector allowing visualization of the complete neuronal morphology. DIV10 GCN, grown in HG conditions, were treated with rotenone applied on the axonal chamber. Morphological integrity of both distal and proximal parts of the axons individualized in the micro-channels was then recorded. As shown in Fig. 4a,b, under these high glucose culture conditions, rotenone treatment did not result in a significant axonal degeneration in control CGN, with very few fragmentation events observed only inside the distal part of the micro-channels. Strikingly, axons from neurons transfected with OPA1^G300E^ (Fig. 4c,d) and DRP1 (Fig. 4e,f) and treated with rotenone, show extensive signs of degeneration in the distal parts of the micro-channel. In DRP1-overexpressing neurons, and to a lesser extent for OPA1^G300E^ expressing neurons, this is associated with further signs of retrograde degeneration toward the proximal part of the micro-channels (Fig. 4c–f) and somatic degeneration in the somatic chamber (not shown). Quantification of the axonal degeneration index in the micro-channels clearly indicates that both DRP1 and OPA1^G300E^ promote a fast retrograde degeneration process (Fig. 4g,h).

Overall, these results showed that modification of mitochondrial dynamics through either impairment of mitochondrial fusion (OPA1^G300E^) or promotion of mitochondrial fission (DRP1) dramatically increased axonal vulnerability toward exogenous stress, suggesting that mitochondrial dynamics is a key player in controlling diffusion of deleterious signals in neuronal axonal cytoplasm towards the cell body.

## Discussion

Increasing research shows that mitochondrial dynamics, which controls mitochondrial morphology and functions, plays a crucial part in the regulation of cellular processes ranging from bioenergetics control to apoptosis related degeneration^20^,^21^,^22^,^23^. As such, mitochondrial functions and dysfunctions are key processes in the context of neurodegenerative diseases. The impairment of mitochondrial transport, respiration and dynamics, have all been shown to be early events in neuronal dysfunction^46^. However, since more than 90% of the neuronal cytoplasm is located in neuronal extensions that extend far from the cell body, focal neurotoxic aggressions (protein aggregates, stroke…) affecting only a portion of neuronal cytoplasm could potentially initiate local degenerative events. Therefore, local control of mitochondrial functions likely plays an important role in gate keeping early events in neuronal degeneration. However, its exact role in the Dying Back retraction process and axonal degeneration remains poorly documented^1^. Cumulatively, our results show that mitochondrial fission is an early and crucial event in axonal degeneration triggered by axonal rotenone application, which only occurs upon local and concomitant glycolytic impairment. Thus, this suggests that unchallenged axons have high axo-protective endogenous signaling capabilities. Artificially forcing mitochondrial dynamics toward fragmentation by impairing fusion or activating fission greatly increases axonal vulnerability toward exogenous stressors like rotenone by itself. Strikingly, increase in mitochondrial fission is also associated with the retrograde spreading of degeneration toward axonal proximal parts. Our results thus suggest that mitochondrial dynamics tightly controls both axonal vulnerability under local stress and the spreading of deleterious signals toward neuronal cell bodies.

Initial works on the role of mitochondrial dynamics in apoptotic processes led to propose a model where shifting its balance towards fusion drives cell resistance to stress whereas cell vulnerability is observed if the balance is shifted towards fission^24^,^27^,^29^,^47^. Surprisingly, in our experimental conditions overexpression of wtOPA1 had no significant consequences on CGN mitochondrial morphology. This may be linked to the fact that CGN already have highly elongated mitochondrial networks when compared to cortical neurons in basal condition. Inactivating fusion or activating fission, through overexpression of OPA1^G300E^ or DRP1 respectively, led to mitochondrial fragmentation without spontaneous apoptosis and axonal degeneration. This is in line with data showing that inducing fragmentation through DRP1 or OPA1 pathways does not systematically promote spontaneous cellular death, a process that depends both on the cell type and cellular stress^27^,^28^,^35^,^47^. The deleterious effects of mitochondrial complex-I inhibitory molecules were previously shown to be inhibited by high glycolytic environments^13^,^48^,^49^,^50^,^51^. Interestingly, our results show that inducing fragmentation through DRP1 activation or OPA1 inactivation leads to axonal degeneration under these protective conditions and may thus indicate that mitochondrial fission in axons reproduces the supplemental stress provided by glycolytic impairment in presence of rotenone. This indeed suggests that the alteration of mitochondrial dynamics and glycolytic impairment may have overlapping consequences resulting in axonal degeneration. It is noteworthy that although OPA1 and DRP1 have different functions, their opposing modulation leading to increased mitochondrial fragmentation with similar consequences on axonal sensitization to apoptosis. Within the mitochondria, OPA1 inactivation destabilizes cristae junctions leading to inner membrane space mobilization of cytochrome *c* as a *bona fide* preparation for apoptosis^52^,^53^,^54^,^55^. DRP1 has also been proposed to play a role in cristae remodeling and could participate in the execution of apoptosis^56^. In addition to a direct impact on apoptosis, the consequences of mitochondrial fragmentation can modify ROS levels^57^,^58^ and/or Ca2+ buffering capacities^59^ together with alterations in energetic states^60^,^61^. Short, fragmented mitochondria may favor an uncoupled state and/or maximal respiration and/or increased protons conductance, leading to a decreased ATP production^62^. Interestingly, while a local decrease in ATP could affect microtubule stability^63^, this was recently shown to lead to axonal fragmentation through a mitochondrial Sirt3 pathway, an enzyme that mitigates rotenone-induced axonal degeneration, as we and others have previously demonstrated^12^,^13^.

In neurons, several studies reported that early mitochondrial fission is triggered by neurodegenerative conditions^36^,^64^ and is linked to an enhanced vulnerability toward apoptosis. In our experimental paradigm of axonal degeneration, pharmacological inhibition of mitochondrial fission by Mdivi-1, which is described to inhibit direct apoptotic signaling through mitochondrial fission and cytochrome *c* release caused by BAX signalization^44^,^45^, protects axons from the deleterious effects of rotenone. This suggests that DRP1 is primarily involved in the early phase of axonal degeneration. While retrograde propagation of axonally initiated insults, coined as the “Dying Back” process, is a common event in neurodegenerative diseases, the mechanisms underlying such retrograde events have been poorly studied in mature CNS neurons. Apoptosis being considered as an autocatalytic event, one could therefore postulate that local activation of pro-apoptotic signaling in axons may lead to a fast retrograde spreading toward the cell body. A surprising observation was that upon axonal rotenone insults in low glucose conditions, although the treated axonal segments degenerate through a caspase-dependent pathway^13^, no significant retrograde spreading toward the cell body was evidenced in basal conditions. Interestingly, while our previous observations suggested that axons have high intrinsic resistance toward direct apoptosis activation^13^,^65^, a recent report showed that direct axonal apoptotic degeneration is gated by a somatic apoptotic signaling^19^. Collectively these findings support the notion that unchallenged parts of the neurons may buffer the spreading of toxic signals in the cytoplasm as previously hypothesized^66^ and suggested by experimental results showing that locally induced mitochondrial damages *in vivo* are due to a local and reversible process^39^. Our results show that pushing mitochondrial dynamics toward fragmentation through an increase of DRP1 activity and, to a lesser extent, by a diminution of OPA1 activity, significantly modifies the spatial degeneration profile triggered by axonal application of rotenone under protective glucose conditions. This therefore experimentally indicates, for the first time, that mitochondrial dynamics is a critical process that allows buffering pathological signal spreading in axons.

This further raises the fundamental question of the spatial spreading of pro-apoptotic signaling modules in the cytoplasm. Some studies have evidenced that, upon apoptosis in cell lines, cytochrome *c* release from mitochondria occurs through spatial waves in the cytoplasm^67^ thus suggesting that apoptosis may occur as an orchestrated spatial pattern. Interestingly, spatial propagation events of mitochondrial membrane permeabilization in cell lines, have been recently shown to be promoted by the dispersion of free radicals leading to the opening of the transition pore^68^. While spreading of degenerative signal may be caused by damaged mitochondria moving to unchallenged, our results showing that rotenone induces a dramatic slowdown of mitochondrial movement and speed are not in favor of that scenario. Interestingly, theoretical studies have shown that spreading of apoptosis signals may occur through diffusion of locally-activated executive apoptotic proteins^69^,^70^. Although not addressing directly these specific questions, our data suggest that mitochondrial integrity is crucial in the axonal diffusion of executive apoptotic factors, as fissionned mitochondria lose their capacity to prevent retrograde propagation of apoptotic factors in axons in the case of intercurrent stress.

Altogether, our data highlight the essential role of mitochondrial dynamics in axonal apoptosis signal amplification. Our results showing that either OPA1 inactivation or DRP1 overexpression is sufficient to overcome specific axonal resistance toward apoptosis by sensitizing mitochondria to sub-threshold rotenone, suggest that mitochondrial priming effect in axons is a key process of axonal degeneration. In the neurodegenerative diseases context where neuronal degeneration can be due to many converging causes^71^, our results could indicate that circumstances inducing mitochondria fragmentation are not sufficient to cause neuronal degeneration. However, reminiscent of progressive ageing process, intercurrent and cumulative events such as protein aggregation, mutation, loss of synaptic signalization could hasten neuronal death and initiate spatial dispersion of locally induced degenerative events.

## Methods

### Microfluidic Chip Production

Microfluidic chips are made up of two elements: 55 mm-high macro-chambers for cell or fluid injection, separated by narrowing arrays of 3 mm-high micro-channels allowing directional axonal outgrowth. The two-compartmented chips were constructed as previously described. Briefly, microfluidic positives masters were produced trough spin coating and UV insulation of SU-8 photolithographic resin on silicon wafers.

Polydimethylsiloxane (Sylgard 184, PDMS, Dow Corning) was mixed with a curing agent (9:1 ratio) and degassed under vacuum. The resulting preparation was poured on microfluidic masters and reticulated at 70 °C for 2 hours. The elastomeric polymer print was detached and two reservoirs were punched manually for each macro-channel. The resulting piece was cleaned with isopropanol and dried. The polymer print and a glass cover slip were treated for 200 seconds in an air plasma generator (100% power, 0.6mBar, Diener Electronic) and bonded together. The chips were placed under UV for 20 minutes and then coated with a solution of poly-D-lysine (10 μg/mL, Sigma) overnight and washed with PBS before cell seeding.

### Primary Neuronal Cultures

Postnatal P5 Swiss mice were purchased from René Janvier (Le Genest Saint Isle, France) and cared for by the animal care facility at University Pierre et Marie Curie (IFR83). Animal care was conducted in accordance with standard ethical guidelines (U.S. National Institutes of Health publication no. 85–24, revised 1985, and European Committee Guidelines on the Care and Use of Laboratory Animals) and the local, IBPS and UPMC, ethics committee approved the experiments. Mouse pups were decapitated and cerebella were micro-dissected. All steps of dissection were conducted in ice cold phosphate buffer saline w/o calcium and Magnesium (DPBS) and supplemented with 0.1% glucose (Life Technologies). Dissected structures were digested with trypsin-EDTA (Life Technologies) for 10 minutes at 37 °C. After tryspin inactivation with Heat Inactivated fetal bovine serum (FBS, PAA), structures were mechanically dissociated in DMEM glutamax (Life Technologies) with plastic pipettes in presence of 10^4^ U/mL DNAse (Sigma Aldrich). After several rinses, cells were re-suspended in DMEM, to a final density of 45 million cells/mL. Cells were then seeded in the somatic compartment by introducing 1.7 μL of the cell suspension into the upper reservoir. Cells flowed into the chamber and adhered within 1–2 minutes. Cell culture medium was then added to the four reservoirs (60 μL/reservoir). Neurons were grown in High Glucose (25 mM) DMEM glutamax containing sodium pyruvate supplemented with streptomycin/penicillin (Thermofisher, Gibco), 10% Fetal Bovine Serum (FBS), N2 and B27 without antioxidant neuronal supplements (Life Technologies). Microfluidic chips were placed in plastic Petri dishes containing H_2_O to prevent evaporation and incubated at 37 °C in a humid 5% C02 atmosphere. The culture medium was renewed six days after seeding. Upon differentiation, 2 or 3 days after seeding, CGN axons entered the micro-channels and reached the second chamber after 5 to 6 days.

### Primary Neurons transfection

OPA1, OPA1^G300E^ or DRP1, and Mito-DsRed plasmids were co-transfected using electroporation according to manufacturer’s recommendation (Microporator, Invitrogen). Briefly, after cell dissociation, cells were washed in PBS centrifuged at low speed. Neurons were re-suspended in electroporation buffer (120 μL for 3 million of cells, Invitrogen) containing 15 μg of plasmids constructs. For co-transfection, a ratio of 2/3 for OPA-1 or DRP-1 plasmids and 1/3 for Mito-DsRed plasmid was used. The electroporator settings were 1350 Volt with 30mseconds for 1 pulse. After the pulse, cells were re-suspended in recovery medium (900 μL of DMEM with 100 μL of FBS). Cells were centrifuged at 1000 rpm for 3 minutes, and were further re-suspended in complete medium and seeded as described above. A transfection efficiency of 40–60% was routinely obtained with a >90% co-transfection efficiency.

### Pharmacological Treatment

All chemicals were prepared as concentrated solutions according to the recommendations of the different manufacturers. Compounds were aliquoted in Eppendorf tubes and used once, to avoid repeated freezing/thawing processes. Aliquots were stored at −80 °C for no longer than two months. Care was taken to protect photosensitive molecules from light by wrapping the test tubes in aluminum foil. Drugs were extemporaneously diluted at their respective final concentration in DMEM containing 10% FBS+ N2+ B27 minus Anti-Oxidant (AO). In order to study the impact of Glucose on rotenone induced toxicity, two distinct DMEM formulations differing only in their glucose concentration were used. HG conditions correspond to DMEM, high glucose, Glutamax Supplement, Pyruvate (Thermofisher, Gibco, Cat ref 10569010) containing 4.5 g.L^−1^ (25 mM) of glucose. LG condition corresponds to DMEM, low glucose, Glutamax Supplement, Pyruvate (Thermofisher, Gibco Cat ref 10567014) containing 1 g.L^−1^ (5 mM) of glucose. Ten days after seeding, Rotenone (5 μM, Sigma-Aldrich) with or without mdivi-1 (20 μM, Tocris) or SP 600125 (10 μM) diluted in LG or HG DMEM were applied for 24 or 48 h according to the treatment. After treatment, neurons were fixed with Paraformaldehyde 4% (Sigma-Aldrich) for 10 minutes at room temperature then washed with PBS.

### Immunofluorescence

Cells were washed twice with D-PBS for 5 minutes and permeabilized for 30 minutes with 0.2% Triton X-100 and 1% BSA in PBS. Primary antibodies PBS solutions were then added and incubated at 4 °C overnight. After, two PBS rinses, cells were further incubated with solutions of the corresponding secondary antibodies for two hours at room temperature. The chips were then rinsed once with PBS and mounted in Mowiol-based medium. The following primary antibodies and dilutions were as follows: alpha-tubulin-FITC (Sigma); Microtubule Associated Protein -2 (Sigma; MAP-2, mouse or rabbit monoclonal (1/500); beta3-tubulin (Sigma; mouse monoclonal 1/500); cytochrome *c* (Cell Signaling 1/300). Species-specific secondary antibodies coupled to Alexa 350, 488, or 555 were used (1/500, Life Technologies,) to visualize bound primary antibodies.

### Image Acquisition

Images were acquired with an Axio-observer Z1 (Zeiss) fitted with a cooled CCD camera (CoolsnapHQ2, Ropert Scientific). The microscope was controlled with Metamorph software (Molecular Imaging) and images were analyzed using ImageJ software (ImageJ, U.S. National Institutes of Health, Bethesda, Maryland, USA).

### Quantification of axonal degeneration and neuronal survival

Axonal degeneration was assessed through beta3-tubulin staining as previously described in ref. 13. Briefly, while beta3-tubulin immunostaining was continuous and homogenous in healthy axons, it appeared punctiform in fragmented axons. The percentage of fragmented axons was calculated by computing the area ratio of circular fragmented axons segments over the total area of axons (six images per axonal chamber). For each condition, cell survival was estimated in the somatic chamber by calculating the percentage of condensed nuclei after Hoechst labeling. Reported values are means of at least three independent experiments, each performed in triplicate. Retrograde axonal degeneration was assessed on CGN co-transfected with a plasmid encoding EGFPN1 (Clontech) and plasmids encoding Empty, OPA1^G300E^ or DRP1. Ten days after electroporation, axonal endings were challenged with 5 μM rotenone for 18 hours. Overlapping images were capture on live neurons, in order to recreate a mosaic image encompassing the somatic, micro-channel and distal areas. Axonal degeneration was assessed by computing the fragmentation index in 2 contiguous micro-channel areas, namely proximal (first half of the micro-channel) and distal (second half of the microchannel).

### Mitochondrial morphology analysis and mitochondria speed analysis

Mitochondrial morphology was assessed from cytochrome *c* or TOM20 fluorescent immunostaining or transfected Mito-DsRed fluorescent signal. Images were acquired using 63X and mitochondrial morphological analysis was performed with Image J. Based on length, mitochondria were grouped in three classes: spherical mitochondria or mitochondria with a size under or equal at 2 μm, filamentous mitochondria between 2 and 4 μm and hyper-filamentous above 4 μm. Mitochondria speed analysis was carried out by video-microscopy with an incubation chamber at 37 °C. We captured 10 minute movies with 12 frames per minute using a 63X oil-objective. Then kymographs are extracted with Image J (plugin Kymograph) and the mitochondria speed is calculated with the kymograph slope.

### Statistical Analysis

Differences were monitored by 1 way ANOVA and a two way ANOVA for mitochondrial size (Fig. 2c), followed by a post-hoc Bonferoni test. For all analyses: *p-value, 0.05; **p-value, 0.01; ***p-value, 0.001.

## Additional Information

**How to cite this article**: Lassus, B. *et al*. Alterations of mitochondrial dynamics allow retrograde propagation of locally initiated axonal insults. *Sci. Rep.* **6**, 32777; doi: 10.1038/srep32777 (2016).

## Supplementary Material

Supplementary Information
