# Corrigendum: Alterations of mitochondrial dynamics allow retrograde propagation of locally initiated axonal insults

**DOI:** 10.1038/srep35328

**Published:** 2016-11-10

**Authors:** Benjamin Lassus, Sebastien Magnifico, Sandra Pignon, Pascale Belenguer, Marie-Christine Miquel, Jean-Michel Peyrin

Scientific Reports 6: Article number: 3277710.1038/srep32777; published online: 09 08 2016; updated: 11 10 2016.

The original version of this Article contained a typographical error in the spelling of the author Sebastien Magnifico, which was incorrectly given as Sebastien Magifico. This has now been corrected in the PDF and HTML versions of the Article.

