# Supplementary material for: Alterations of mitochondrial dynamics allow retrograde propagation of locally initiated axonal insults
Source: Sci Rep. 2016 Sep 8;6:32777. doi: 10.1038/srep32777 (PMC5015069; doi:10.1038/srep32777)
Supplement: Supplementary Information [file srep32777-s1.pdf]

Alterations of mitochondrial dynamics allow retrograde propagation of locally initiated axonal insults

Lassus B[1,2], Magnifico S[1,2], Pignon S[1,2], Belenguer P[3], Miquel MC[3*] and Peyrin JM[1,2*]

Supplementary Figure 1: GFP-transfected CGN (GFP$^+$-CGN) seeded in microfluidic chambers. Neuronal somas are positioned in the somatic chamber **(left),** their axons growing in the micro-channels **(center, between yellow dotted lines)** and reaching the axonal chamber **(right). (a)** Typical 10 Days In Vitro (DIV) GFP$^+$-CGN grown in low glucose medium (LG) in both chambers, 48 hours post treatment axonal with sham control medium. **(b-c)** Ten In Vitro (DIV) GFP$^+$-CGN grown in low glucose medium 24h **(b)** or 48h **(c)** post axonal rotenone exposure (5µM). Note that application of rotenone in the axonal chamber led to axonal degeneration in the axonal (treated) chamber with a slow and limited retrograde degeneration in the distal part of micro-channel area after 48 hours (white arrow).

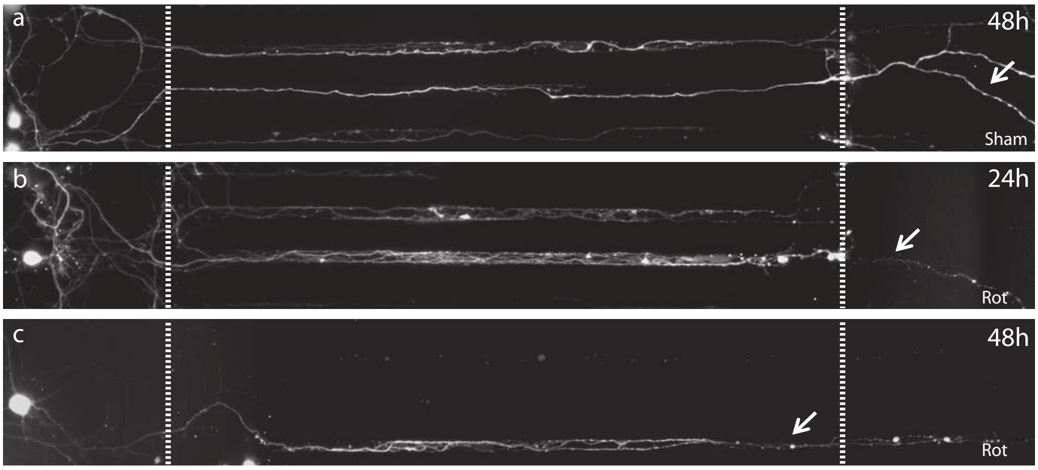

a
48h
Sham
b
24h
Rot
c
48h
Rot
